# Supplementary material for: Development and Evaluation of a Novel HNB Based Isothermal Amplification Assay for Fast Detection of Pyrimethamine Resistance (S108N) in Plasmodium falciparum
Source: Int J Environ Res Public Health. 2019 May 10;16(9):1635. doi: 10.3390/ijerph16091635 (PMC6539687; doi:10.3390/ijerph16091635)
Supplement: Supplementary file 1 [file ijerph-16-01635-s001.pdf]

Supplementary file

Development and evaluation of a novel HNB based isothermal amplification assay for fast detection of Pyrimethamine resistance (S108N) in *Plasmodium falciparum*

Madhvi Chahar *, Anup Anvikar and Neena Valecha

Division of Epidemiology & Clinical Research, National Institute of Malaria Research, Sector-8 Dwarka, New Delhi 110077, India; (A.A.) anvikar@gmail.com ; (N.V.) neenavalecha@gmail.com

(M.C.)* Correspondence: madhvi23rana@gmail.com

Correspondence and requests for materials should be addressed to M.C.

Table S1. List of *P. falciparum* samples used in the repeatability test of SNP-LAMP for the fast detection of pyrimethamine resistant mutants (S108N) and their corroboration by gold standard sequencing method.

Serial No.	*P. falciparum* samples	Region	Resistance profile [a]	HNB LAMP [b]	Sequencing [c]
1	RI-01	Ranchi, India	PYR^M	+	+
2	RI-02	Ranchi, India	PYR^M	+	+
3	RI-03	Ranchi, India	PYR^W	-	-
4	RI-04	Ranchi, India	PYR^W	-	-
5	RI-05	Ranchi, India	PYR^W	-	-
6	RI-06	Ranchi, India	PYR^W	-	-
7	RI-07	Ranchi, India	PYR^M	+	+
8	RI-08	Ranchi, India	PYR^W	-	-
9	RI-09	Ranchi, India	PYR^W	-	-
10	RI-10	Ranchi, India	PYR^W	-	-
11	RI-11	Ranchi, India	PYR^M	+	+
12	RI-12	Ranchi, India	PYR^W	-	-
13	RI-13	Ranchi, India	PYR^M	+	+
14	RI-14	Ranchi, India	PYR^W	-	-
15	RI-15	Ranchi, India	PYR^W	-	-
16	RI-16	Ranchi, India	PYR^W	-	-
17	RI-17	Ranchi, India	PYR^W	-	-
18	RI-18	Ranchi, India	PYR^W	-	-
19	RI-19	Ranchi, India	PYR^M	+	+
20	RI-20	Ranchi, India	PYR^W	-	-
21	RI-21	Ranchi, India	PYR^W	-	-
22	RI-22	Ranchi, India	PYR^W	-	-
23	RI-23	Ranchi, India	PYR^W	-	-
24	RI-24	Ranchi, India	PYR^M	+	+
25	RI-25	Ranchi, India	PYR^M	+	+
26	RI-26	Ranchi, India	PYR^W	-	-
27	RI-27	Ranchi, India	PYR^W	-	-
28	RI-28	Ranchi, India	PYR^M	+	+
29	RI-29	Ranchi, India	PYR^W	-	-
30	RI-30	Ranchi, India	PYR^W	-	-
31	RI-31	Ranchi, India	PYR^W	-	-

32	RI-32	Ranchi, India	PYR^{M}	+	+
33	RI-33	Ranchi, India	PYR^{W}	-	-
34	RI-34	Ranchi, India	PYR^{W}	-	-
35	RI-35	Ranchi, India	PYR^{M}	+	+
36	RI-36	Ranchi, India	PYR^{M}	+	+
37	RI-37	Ranchi, India	PYR^{W}	-	-
38	RI-38	Ranchi, India	PYR^{W}	-	-
39	RI-39	Tripura, India	PYR^{W}	-	-
40	RI-40	Ranchi, India	PYR^{M}	+	+
41	RI-41	Ranchi, India	PYR^{W}	-	-
42	RI-42	Ranchi, India	PYR^{M}	+	+
43	RI-43	Ranchi, India	PYR^{W}	-	-
44	RI-44	Ranchi, India	PYR^{W}	-	-
45	RI-45	Ranchi, India	PYR^{M}	+	+
46	RI-46	Ranchi, India	PYR^{M}	+	+
47	RI-47	Ranchi, India	PYR^{M}	+	+
48	RI-48	Ranchi, India	PYR^{W}	-	-
49	RI-49	Ranchi, India	PYR^{M}	+	+
50	RI-50	Ranchi, India	PYR^{W}	-	-
51	RI-51	Ranchi, India	PYR^{W}	-	-
52	RI-52	Ranchi, India	PYR^{W}	-	-
53	RI-53	Ranchi, India	PYR^{M}	+	+
54	RI-54	Ranchi, India	PYR^{W}	-	-
55	RI-55	Ranchi, India	PYR^{M}	+	+
56	RI-56	Ranchi, India	PYR^{W}	-	-
57	RI-57	Ranchi, India	PYR^{W}	-	-
58	RI-58	Ranchi, India	PYR^{W}	-	-
59	RI-59	Ranchi, India	PYR^{M}	+	+
60	RI-60	Ranchi, India	PYR^{W}	-	-
61	RI-61	Ranchi, India	PYR^{W}	-	-
62	RI-62	Ranchi, India	PYR^{M}	+	+
63	RI-63	Ranchi, India	PYR^{M}	+	+
64	RI-64	Ranchi, India	PYR^{W}	-	-
65	RI-65	Ranchi, India	PYR^{W}	-	-
66	TR-66	Ranchi, India	PYR^{M}	+	+
67	TR-67	Ranchi, India	PYR^{W}	-	-
68	TR-68	Ranchi, India	PYR^{M}	+	+
69	TR-69	Ranchi, India	PYR^{M}	+	+
70	TR-70	Ranchi, India	PYR^{M}	+	+
71	TR-71	Ranchi, India	PYR^{W}	-	-
72	TR-72	Ranchi, India	PYR^{W}	-	-
73	TR-73	Ranchi, India	PYR^{W}	-	-
74	TR-74	Ranchi, India	PYR^{M}	+	+
75	TR-75	Ranchi, India	PYR^{M}	+	+
76	TR-76	Ranchi, India	PYR^{W}	-	-
77	TR-77	Ranchi, India	PYR^{M}	+	+
78	TR-78	Ranchi, India	PYR^{W}	-	-
79	TR-79	Ranchi, India	PYR^{W}	-	-
80	TR-80	Ranchi, India	PYR^{M}	+	+
81	TR-81	Ranchi, India	PYR^{W}	-	-
82	TR-82	Ranchi, India	PYR^{M}	+	+
83	TR-83	Ranchi, India	PYR^{W}	-	-
84	TR-84	Ranchi, India	PYR^{M}	+	+
85	TR-85	Ranchi, India	PYR^{W}	-	-
86	TR-86	Ranchi, India	PYR^{W}	-	-
87	TR-87	Ranchi, India	PYR^{W}	-	-
88	TR-88	Ranchi, India	PYR^{M}	+	+
89	TR-89	Ranchi, India	PYR^{M}	+	+
90	TR-90	Ranchi, India	PYR^{W}	-	-
91	TR-91	Ranchi, India	PYR^{W}	-	-

92	TR-92	Ranchi, India	PYR^M	+	+
93	TR- 93	Ranchi, India	PYR^W	-	-
94	TR-94	Ranchi, India	PYR^M	+	+
95	TR-95	Ranchi, India	PYR^W	-	-
96	TR-96	Ranchi, India	PYR^W	-	-
97	TR-97	Ranchi, India	PYR^M	+	+
98	TR-98	Ranchi, India	PYR^W	-	-
99	TR-99	Ranchi, India	PYR^M	+	+
100	TR-100	Ranchi, India	PYR^M	+	+
A*	3D7	Malaria parasite bank	PYR^W	-	-
C*	DD2	Malaria parasite bank	PYR^M	+	+

[a], PYR^M and PYR^W state that the *P. falciparum* sample is mutant(S108N) and wild type to pyrimethamine respectively. [b] and [c] '+' an '– ' indicate the positive and negative results by SNP-LAMP assay and gold standard Sequencing. * Specifies the wild type & mutant control of *P. falciparum.*
